# Autonomy in Japan: What does it Look Like?

**DOI:** 10.1007/s41649-022-00213-6

**Published:** 2022-08-11

**Authors:** Akira Akabayashi, Eisuke Nakazawa

**Affiliations:** 1 Department of Biomedical Ethics, Faculty of Medicine, University of Tokyo, Tokyo, Japan; 2 Division of Medical Ethics, New York University School of Medicine, New York, NY USA

**Keywords:** Autonomy, Japan, Family, Cross-cultural, Dialogue, Global bioethics

## Abstract

This paper analysed the nature of autonomy, in particular respect for autonomy in medical ethics/bioethics in Japan. We have undertaken a literature survey in Japanese and English and begin with the historical background and explanation of the Japanese word *Jiritsu (autonomy)*. We go on to identify patterns of meaning that researchers use in medical ethics / bioethics discussions in Japan, namely, Beauchamp and Childress’s individual autonomy, relational autonomy, and O’Neill’s principled autonomy as the three major ways that autonomy is understood. We examine papers discussing these interpretations. We propose using the term ‘a form of autonomy’ first used by Edmund Pellegrino in 1992 and examine the nature of ‘a form of autonomy.’ We finally conclude that the crux of what Pellegrino calls ‘something close to autonomy,’ or ‘a form of autonomy' might best be understood as the minimization of physician paternalism and the maximization of respect for patient preference. Simultaneously, we introduce a family-facilitated approach to informed consent and respond to criticism by Laura Sullivan. Finally, we discuss cross-cultural approaches and global bioethics. Furthermore, we use the term ‘Bioethics across the Globe’ instead of ‘Global Bioethics’, calling for international scholars to write works to provide an in-depth understanding of each country. We conclude that deep understanding of others is pivotal for dialogue to be of value. We hope this article will deepen the reader’s understanding of Japan and will contribute to the progress of bioethics worldwide.

## Introduction

The overarching term ‘autonomy’ emerged in the field of medical ethics/bioethics in the 1970s, when this area of scholarship was developed. Starting in the USA, the field has spread worldwide. Bioethics emerged in Japan in the 1980s. However, foreign researchers have frequently asked Japanese researchers how autonomy is understood in their country. One of the reasons for this is that there are only a small number of English publications from Japan. Thus, we examine the concept of ‘autonomy’ in Japan in the hope that this article will give the readers greater clarity and insight about the meaning of autonomy in Japan.

## Historical Background

Autonomy is a concept used outside the field of medical ethics / bioethics. It will assist readers to introduce Huang’s (2018) article titled ‘[T]he concept of “self-government” across cultures: From the Western World to Japan and China’. This article describes how Western ideas were introduced into Japan that also relate to bioethics (Huang 2018, 54):

*In the mid-nineteenth and early twentieth century, the Western concept of “self-government” was introduced to East Asia along with the spread of European culture. In Japan and China, this concept confronted traditional beliefs and norms dictating these two countries and thus transformed as it was translated, circulated, and institutionalized, not unlike “freedom”, “democracy”, “constitutionalism”, and other notions.*

*Jichi*, the Japanese translation of ‘self-government’, is closely related to ‘freedom’ (*Jiyu*) and ‘democracy’ (*Minshu-shugi*). These terms were created alongside other neologisms that conveyed Western concepts. However, these new phrases were by no means modern inventions but originate from ‘wasei-kango’ (Japanese-made Chinese words), a body of words developed from Chinese. We assume that ‘autonomy’ was translated into *Jiritsu* (自律) because *Ji* (自) means ‘self’ (autos) and *Ritsu* (律) means ‘rule’ or ‘law’ (nomos). Although some discussions are still ongoing on how to translate the term autonomy into Japanese, the majority of people in Japan use *Jiritsu*.

As Huang (2018, 61) has stated:

*“Self-government,” in this sense, referred to the capability of self-management, drawing much inspiration from the ideal of “self-cultivation” in Confucianism. Nevertheless, it intended to foster “the spirit of autonomy” and was therefore quite different from its Confucian counterpart.*

## What Kinds of Autonomy are there in Japan?

In what follows, we focus on the discussions in the field of medical ethics/bioethics in Japan. We performed a database search using PubMed, Philpaper, and Philosopher’s index for English literature, *Ichushi*, J-stage, and Medical-online for Japanese literature. The search keywords used were *autonomy*, *Japan*, and *relational autonomy*. There were more than 500 hits, and we went through all abstracts. We then classified the papers and books according to the patterns in which autonomy is used. Although this search was not exhaustive, we think that it is sufficient to lead us to a meaningful conclusion.

In addition, there was discussion about when concept of autonomy and its centrality first emerged in Japanese bioethics. Some believed that that Japan did not need to embrace the concept of respecting autonomy at all, since it was a Western idea. This position is no longer popular.

### ‘Respect for Autonomy’: Beauchamp and Childress

The most popular and frequently used term, especially by healthcare professionals and in health-related papers, is ‘respect for autonomy’ as discussed by Beauchamp and Childress (2019), which is based upon the idea of ‘individual autonomy’. Beauchamp and Childress (2019, 99) do not define ‘autonomy’ itself but describe autonomous action thus: “The autonomous individual acts freely in accordance with a self-chosen plan, analogous to the way an autonomous government manages its territories and sets its policies”.

In Japan, the word autonomy is frequently used without definition. Because only the concept of Beauchamp and Childress’s autonomy was imported into Japan in the 1990s, most Japanese people do not know that there are other types of autonomy.

Therefore, if the author of a paper uses autonomy or *Jiritsu* without any specific commentary referring to the English or Japanese literature, it is assumed that the version of autonomy being referred to is that developed by Beauchamp and Childress (Ruhnke et al. 2000; Tsuruwaka et al. 2020; Tanaka and Kodama 2020). In the field of psychology, this trend seems to be the same (Yu et al. 2018; Tan et al. 2021).

This formulation of autonomy has engendered much criticism in Japan as well as in Western scholarship. The authors who formulated these theories have responded in defence of their definition. For example, Childress (1990, 12) states that one of the reasons is misdirected criticism. He claims: “In several ways, the principle of respect for autonomy has been misunderstood and misinterpreted, in part as a result of flawed formulations and defenses by its supporters”. Childress (1990, 17) concludes:

*Yes, we should go beyond the principle of respect for autonomy - in the sense of going beyond its misconceptions and distortions and in the sense of incorporating other relevant moral principles. But going beyond should not mean abandoning. Despite its complexity in application, despite its limits in scope or range and in weight or strength, and despite social changes, the principle of respect for personal autonomy has a critical role to play in biomedical ethics in the 1990s. But that role requires a sense of limits; we must not overextend or overweight respect for autonomy.*

Japan is no exception in its use of this formulation, and even official bodies such as the Ministry of Health, Labor, and Welfare and the Japan Medical Association use this version of autonomy without defining it.

In order to understand Japan further in this context, we want to introduce an article by Asai et al. (2022), which discusses obstacles to clinical shared decision-making. Asai et al. (2022, 138) state that “The situation is complicated further by differences in the various understanding of personal autonomy […]. At least two kinds of autonomy are at play—individualistic autonomy and relational autonomy.” Although their focus is ‘shared decision-making,’ the paper introduces many crucial issues which Japan’s medical ethics/bioethics faces at present. It may be also informative to look at the article by Childress and Childress (2020) regarding shared decision-making in the USA.

### Relational Autonomy

Relational autonomy *(translated as Kankeiteki-Jiritsu)* is the second most popular term used in papers by Japanese scholars. Since Japan is regarded as a family-oriented society, proponents of relational autonomy often emphasise that autonomy created by individualistic Western countries fits poorly with issues related to medical ethics and bioethics in Japan. Thus, in the fields of nursing, medical treatment and care, palliative care, and other end-of-life issues such as advance care planning (ACP), relational autonomy is highly relevant and used commonly (Brandi and Naito 2006; Morita et al. 2020; Akiba 2021). However, as in the case of Beauchamp and Childress’s respect for autonomy, in the discussion of relational autonomy, too, no working definition is usually given.

We found one recent philosophical paper related to relational autonomy in Japan, written by a Japanese researcher (Asagumo 2021, 57). In her abstract, she suggests that “the concept of relational autonomy might have some practical and valuable implications in a country where individual autonomy is considered incompatible with societal values.”

As Asagumo (2021, 61) correctly states, “[R]elational autonomy denotes various perspectives that understand autonomy from a relational standpoint”. However, Asagumo uses the definition of relational autonomy proposed by Gomez-Virseda et al. (2020) as follows (Asagumo 2021, 61):

*Based* *on* *this* *systematic* *review*, *Gomez-Virseda* *et* *al*. *(*2020*, 3–5)* *identify* *four* *shortcomings* *of* *the* *traditional* *understanding* *of* *autonomy* *from* *which* *a* *key* *understanding* *of* *relational* *autonomy* *can* *be* *developed:* *autonomy* *entails* *more* *than* *merely* *possessing* *cognitive* *capacity;* *autonomy* *is* *not* *exercised* *by* *patients* *existing* *in* *a* *social* *and* *cultural* *void;* *autonomy* *is* *not* *a* *binary* *‘all-or-nothing’* *condition; autonomy is* *not* *exercised* *in* *terms* *of isolated* *discrete* *discussions*. *Autonomy* *is* *a* *multidimensional* *capacity* *which* *consists* *of* *emotions* *and bodily* *mediated experiences*  *besides rationality;* *autonomy is* *exercised in a* *sociocultural context* *that shapes us, and the relationships between* *patients, family, and personal relationships, and healthcare professionals are able to* *enhance or undermine autonomy;* *for these reasons, autonomy manifests itself* *in a scale, and we can be more or less* *autonomous rather than be or not be autonomous;* *therefore, autonomy is a temporal perspective evolving and unfolding* *over time through interactions with others.* *I argue that it would be beneficial to introduce this* *analysis of autonomy into clinical practice in Japan. I defend my view in relation to the second, third, and fourth points of the* *shortcomings of individual autonomy suggested by Gomez-Virseda et al.*

Thus, Asagumo uses the almost same definition proposed by researchers in a Western country (i.e., Belgium). Asagumo (2021, 61) argues that it would be beneficial to introduce this analysis of autonomy into clinical practice in Japan.

As Japan is a family-oriented society, we presume that the relational character of clinical decision-making already prevails. Asagumo’s (2021, 67) conclusion, “[A] change in the understanding of autonomy in medicine could pave the way for fulfilling patients’ wishes in Japan”, is somewhat confusing to us. We ask the question: are patients’ wishes not fulfilled in Japan?

The Japanese philosopher, Seisuke Hayakawa, who teaches relational autonomy at the University of Tokyo, Faculty of Literature says, ‘*I have laid more emphasis on empathic interaction necessary for the development of relational autonomy (or agency) and its attendant trusting relationship.*’ (Personal communication).

We move to Asagumo’s discussion on advance directives (ADs). She (Asagumo 2021, 61) states “a more fundamental rethinking of autonomy with greater attention given to the concept of relationality might help to facilitate better understanding and realization of AD in Japan”. ADs are not legally binding in Japan. Questionnaire surveys in Japan in 1996 and in 1998 (Akabayashi et al. 1997, 2003) and articles produced by international collaborations on ADs (Sass et al. 1996; Voltz et al. 1998) may be useful to understand why this is so. Many Japanese respondents to the survey did not like written forms of ADs. Japanese respondents also preferred entrusting decision-making to their families in case of an emergency (see also Sehgal et al. 1992). After 25 years, ADs remain uncommon in Japan. Moreover, there are three or four types of ADs. The Japan Society for Dying with Dignity has created a form for AD (Living Will). This is the first of its kind, but it does not seem sufficient for use in the clinical setting. The AD consists of two components, namely ‘designation of durable power of attorney’ and ‘direction to healthcare professionals.’ From a relational autonomy perspective, which component is suitable for the Japanese context? Or are they both suitable? In addition, as Asagumo correctly points out, it is difficult to predict the future or imagine when one is dying; that is, there is a theoretical limitation in ADs. Proxies also have limitations to predict patients’ real wishes (Akabayashi et al. 1997, 2003; Emanuel 1993).

Recently, the Ministry of Health, Labour, and Welfare in Japan has been promoting the Advanced Care Plan (ACP), a concept proposed in the late twentieth century in the USA (Prendergast 2001; Teno et al. 1994). Despite several efforts to evolve new ACP styles (Martin et al. 2000; Johnstone and Kanitsaki 2009), the ACP has not been easily implemented in Japanese clinical settings. Although it has been reported that the ACP is now prevalent in Asian countries (Cheng et al. 2020), it is unclear whether rethinking autonomy will facilitate ADs or the ACP in Japan because it is absolutely socio-cultural reasons as stated above. Many Japanese did not like written forms of ADs. Japanese also preferred entrusting decision-making to their families in case of an emergency. This expression of autonomy in Japan will not change easily.

In what follows we would like to discuss articles that explain why it is still not a common practice to withdraw ventilators from end-of-life patients in Japan, and why physicians still fear being sued (Nakazawa et al. 2019a, b). In these articles, the authors explain the related concepts of dependency (*amae*) and village society and critically argue that Japanese people are hesitant to make decisions on this matter. We believe that *amae* is one of the factors. *Amae*, which means dependency, is closely associated to the idea of relationality.

### Onora O’Neill’s Conception of Autonomy

Onora O’Neill’s (2002) criticises the contemporary conception of autonomy in medical ethics/bioethics and proposes an alternative interpretation derived from Immanuel Kant, which she calls ‘principled autonomy’. In the first chapter of her book titled ‘Gaining autonomy and losing trust?’, O'Neill (2002, 2-4) writes as follows:

*During these years no themes have become more central in large parts of bioethics, and especially in medical ethics, than the importance of respecting individual rights and individual autonomy. [...] Yet [...] public trust in medicine, science and biotechnology has seemingly faltered. [...] Is some loss of trustworthiness and of trust an acceptable price for achieving greater respect for autonomy? Do we have to choose between respect for individual autonomy and relations of trust? None of these prospects would be particularly welcome: we prize both autonomy and trust. Yet can we have both?*

As mentioned above, O’Neil touched on the mistrust of medicine, which was also prevalent in Japan during the time that she was writing. This ‘mistrust of medicine’ may be one of the factors for philosophers in Japan to engage with O’Neil’s (2002) concept of autonomy. As well, O’Neill (2003) and Japanese philosophers alike were not satisfied with the way informed consent was both discussed and utilised.

This is exemplified by Enzo et al. (2021, 41) who refer to O’Neill in their public prenatal screening paper, stating, “we will focus on O’Neill’s argument about rights and obligations. Drawing on her position, we will show that it is important to change our normative perspective to obligations and to explore government obligations concerning respect for autonomy”. It is also worth noting that they also state (Enzo et al. 2021, 44), “Therefore, in addressing our research questions, we need to change our normative perspective from rights (in particular that of autonomy) to obligations, and to explore government obligations concerning the promotion of autonomy.”

### Other Papers

Finally, we introduce three more papers written in Japanese. These draw on the stream of philosophy known as communitarianism. As Miller (2014, 306) notes, criticism of autonomy was also launched from a communitarian perspective (Sandel 1982; Callahan 1984). Communitarians object to liberal individualism on several grounds. Central is the claim that the socialisation process determines or shapes the value and preferences of individuals (Miller 2014, 306). Sasaki’s position is close to this. Sasaki (1998) argues for a concept known as ‘collective autonomy’ (authors’ translation of *Kyodouteki Jiritsu*). Another one is Hoshikawa’s (1994) ‘die autonome Offentlichkeit’ (autonomous publicity: the authors’ translation of *Jiritsuteki Kyodousei*)*,* relying mainly on Jürgen Habermas’s idea. Judai (2014) argues that for a dying person, it is more important for caretakers to share the suffering with the patient than for the patient to be ‘autonomous’. This argument is drawn from the work of Lois Shepherd (1996) and Daryl Pullman (2002).

## Enzo et al.’s Challenge

In 2019, Enzo et al. published an article on the re-examination of respect for autonomy in the context of the management of chronic conditions. We believe that this paper is the most robust philosophical argument in the clinical/medical ethics setting, amongst the literature that we found. The authors are aware that the number of patients with chronic conditions is rising globally, and biomedical ethicists are interested in the ethical issues raised by these diseases.

Enzo et al. (2019, 86-87) were not satisfied with the contemporary accounts of autonomy and stated that “we can see a radical change in the interpretation of this principle (hereafter, called “new accounts of respect for autonomy”): respect for autonomy is not interpreted as a deontological principle any longer. [...] New accounts of respect for autonomy underpin many public programmes and policies worldwide that affect both chronic disease management and health promotion. […] However, little attention has been paid to the risk of applying new accounts of autonomy in clinical settings.”

Enzo et al. (2019, 90) later developed their arguments. First, they rejected the new accounts of respect for autonomy and criticised modern Kantian philosophers’ accounts in healthcare settings. Then, they suggested eliminating the concept of autonomy from the formulation of the basic principle that commands us to respect persons and proposed ‘respect for persons’ as an alternative basic principle. They were careful to differentiate their concept from that proposed for research ethics in the Belmont Report. They counter argued possible criticisms and emphasised that their argument might be interpreted as providing guidelines.

This important work nonetheless raised questions for us for further clarification and discussion, namely:

1. If the authors eliminate and replace ‘autonomy’ with an alternative term ‘respect for persons’, questions occur on what this means.
2. In a subsequent paper (Enzo et al. 2021, 44) on public prenatal screening, they state, “This does not mean, however, discarding the concept of autonomy, which is a dominant value today”. Is this argument inconsistent with their earlier paper?
3. The authors focus on clinical settings. Do we need another principle for research ethics argument? (Johnston and Zacharias 2017).
4. In the counterargument, Enzo et al. (2021, 93) state, “We respond to such criticism, by emphasising the importance of shared decision-making by all parties, including medical professionals and patients’ families”. What is the authors’ account of shared decision-making?
5. Padela et al. (2015) argued that the principle of ‘respect for persons’ informed by culture-specific ideas of personhood may offer an improved ethical construct for analysing and guiding medical practice in a globalised and plural world. How are the Enzo et al.’s ‘respect for persons’ and Padela et al.’s accounts different?

## ‘A Form of Autonomy’ or ‘Something Close to Autonomy’

Scholarly discussions on the nature of autonomy in medical ethics/bioethics have a long history. This was sparked by North American bioethicist Edmund Pellegrino (1992, 1735) in his editorial in the* Journal of the American Medical Association (JAMA*). He first used the phrases ‘a form of autonomy’ and ‘something close to autonomy’:

*In many cultures clinicians encounter patients who are fully aware of the gravity of their condition but choose to play out the drama in their own way.* *This may include not discussing the full or obvious truth.* *This is a form of autonomy, if it is implicitly and mutually agreed on,* *between physician and patient.*

He continued:

*Autonomy is still a valid and universal principle because it is based on what it is to be human. The patient must decide how much autonomy he or she wishes to exercise, and this amount can vary from culture and culture. It seems probable that the democratic ideals that lie behind the contemporary North American concept of autonomy* *will spread and that something close to it will be the choice of many individuals in other countries as well.*

In response, Antonella Surbone (1992, 1662) described her dilemma when trying to apply the ideals of medical ethics she learned in the USA to her native Italy, where autonomy is often translated as isolation. She also argued that a better insight into the motivations for existing differences in truth-telling to patients with cancer can be achieved by understanding the dynamic provisional nature of truth and the relational nature of autonomy’ (Surbone 2006). Further arguments added by Akabayashi et al. (1999, 299) are as follows.

*[W]hen considering this issue in the international context, the term “autonomy” should be used carefully since it is not a concept with only one meaning. Pellegrino does not specify whether his notion of a North American concept of autonomy refers to the definition of autonomy or the degree of exercise of autonomy, or both. Surbone’s remark that autonomy is often synonymous with isolation in Italy illustrates that the exercise of autonomy differs in Italy and North America.*

Pellegrino did not exactly define or give a detailed explanation of ‘a form of autonomy’ or ‘something close to autonomy’. However, his definition is still valuable today, after 30 years. Therefore, the fundamental question remains: What does ‘a form of autonomy' look like in other countries?.

In Japan, the nature of autonomy has been a subject of much discussion. Hereinafter, we use the term ‘a form of autonomy’ instead of ‘something close to autonomy’ because the former is closer to the expression used to explain autonomy linguistically, for example, relational autonomy or principled autonomy. However, we assume that Pellegrino uses ‘a form of autonomy’ and ‘something close to autonomy’ in the same way.

## Criticism of the Family-Facilitated Approach: an Example of ‘a Form of Autonomy’

This section of our paper brings together the earlier sections.

Among the articles published on this topic, we recommend the following, all written in English (Akabayashi et al. 1999; Akabayashi and Slingsby 2006; Akabayashi and Hayashi 2014a, b; Becker 2014; Fan 2014; Ho 2014). Akira Akabayashi described an example of a form of autonomy in order to understand the nature of what Pellegrino said in 1992. Following this, Akira Akabayashi and Brian T. Slingsby (2006, 11) proposed a family-facilitated approach:

*A family-facilitated approach to informed consent where the family and the patient function as a single unit differs from the more popular first-person approach. In this paper, we define a family-facilitated approach as a process of informed consent in which a patient’s family communicates with the attending physician and medical staff and often makes treatment-related decisions. This differs from acting as a proxy in that the patient does not officially appoint his or her family. Family-facilitated decision making thus rests on the premise that a patient–family fiduciary relationship exists and that the patient identifies his or her self more as a component of the family unit than as an independent individual.*

Akabayashi and Slingsby (2006, 13) continued:

*Moreover, a family-facilitated approach does not necessarily contradict with the general ethical principle of respect for autonomy in the United States. In fact, a family-facilitated approach to informed consent may be respecting a patient’s individual choice—that is, if a patient who holds an interdependent view has a propensity to prefer a family-facilitated approach, providing this approach to informed consent may indeed be respecting patient autonomy.*

Following this *American Journal of Bioethics* (AJOB) article, Akira Akabayashi and Yoshinori Hayashi (2014a, 742) published a concluding assertion in a book chapter on *The Future of Bioethics*. They concluded:

*To see why we believe a family-facilitated approach is compatible with patient autonomy, let us revisit our conclusions from our analysis of Case 2. In Case 2, the argument was: if a patient who holds an interdependent view has a propensity to prefer a family-facilitated approach, providing this approach to informed consent may indeed be respecting patient autonomy. The line of reasoning behind this argument begins with our assumption that patient autonomy is being respected when a patient’s preferences are fulfilled. We then argue that a patient with an inter-dependent view of himself is highly likely to be more comfortable with a family-facilitated approach, thus taking a family-facilitated approach is consistent with the patient’s preference. Therefore, we conclude, a family-facilitated approach is consistent with the patient’s autonomy in that it is in accord with the patient’s preferences.*

Akabayashi and Hayashi (2014a, 744-745) continue:

*In what sense can we claim that the family-facilitated approach is still compatible with patient autonomy? […] in the family-facilitated approach, physician paternalism and any undue influence from the family are excluded as points of contention. In the family-facilitated approach, the patient’s desire for family decision-making, authorized by tacit consent, is respected, and the possibility that the physician will make decisions against the will of the patient is removed. *

*It is true that strong family involvement in medical decision-making may appear oppressive and in conflict with patient autonomy. However, in the family-facilitated approach, it is the patients who want their family to make the medical decisions, as they see themselves first as part of a family unit, and family decision-making is preferred by the patients themselves. Therefore, family involvement is not an undue restriction to patient autonomy. *

*Based on these considerations we conclude that the family-facilitated approach is compatible with the motive behind the conventional view of autonomy, although the family-facilitated approach is not compatible with autonomy in the strictest conventional sense of the word. However, we maintain that it is consistent with some particular sort of autonomy, which fits well with the Japanese clinical settings where the family’s role in treatment choice is considered more significant. […] This is congruent with Pellegrino’s expression “a form of autonomy.” Pellegrino, who first stated that a form of autonomy used in the case of a patient in Italy, did not offer a clear definition.* ***We claim that the crux of what Pellegrino calls “something close to autonomy,” “a form of  autonomy” might best be understood as the minimization of physician paternalism and respect for patient preference.*** *(Authors’ emphasis)*

Through this series of three informed consent articles, Akira Akabayashi (AA) has attempted to elucidate what ‘a form of autonomy’ looks like in Japan. There is, nonetheless, wide debate about our position (Nagai 2017). Laura Specker Sullivan (2016a), for example, typifies the robust criticism many Western researchers have to our arguments.

## Criticism by Sullivan

Sullivan’s (2016a) criticisms are set out in her article in the *Kennedy Institute of Ethics Journal*. We will respond to her criticisms as she sets them out.

In the introduction, Sullivan (2016a, 47) wrote:

*[…], at times, disagreement on particular issues becomes so entrenched that understanding seems impossible. In such circumstances, how might bioethicists proceed? In answering this question, this paper considers a particularly significant area of disagreement: the informed consent standards for medical practices in different countries. Since many medical procedures are transnational, it is thought that the informed consent standards for* ***these procedures should be universal.*** *(Author's emphasis)*

It seems that Sullivan is a universalist. Sullivan (2016a, 55) summarised AA’s position as follows:

*In short, Akabayashi values the possibility of dialogue about ethical valuations across cultures, so he does not allow that ethical judgments are predicated on unique cultural values. Rather, ethical justification must have a universal foundation such that individuals in different cultures can engage in dialogue and critique. For Akabayashi, this foundation is a set of common abstract ideals used in moral judgment and justification. While judgment and justification occur in the context of local realities, their content (i.e., Beauchamp and Childress’s four principles: autonomy, beneficence, nonmaleficence, and justice) and methodology (i.e., application of principles) are universal […]*

*Akabayashi recognizes the efforts of Ruiping Fan to ground family-oriented medical practice in Confucian culture as similar to his attempt to accommodate the importance of the family into medical decision-making. Yet he ultimately concludes that Fan’s justification, which relies on particular Chinese or East Asian cultural concepts “incommensurable with the Western principle of autonomy” (*Akabayashi and Hayashi 2014a*, *b*, 747), differs from his own, which seeks consistency or compatibility with Western autonomy. For Akabayashi, commensurability of ethical concepts across cultures is necessary for successful cross-cultural dialogue, so his defense of the Japanese practice of informed consent in terms of a form of autonomy compatible with Western autonomy achieves two goals: it accounts for the practice in nonculturally relative terms, and it justifies the practice according to nonculturally relative standards.*

However, there is some misunderstanding here, AA is labelled a universalist and Ruiping Fan a relativist. Let us start by looking at her criticism of AA (Sullivan 2016a, 58, 60, 61, 62, 63, 67, 69, and 73.

1. *The shared goal of Akabayashi’s and Fan’s arguments is to justify their cultures’ practices. Both assume that ethical justification must proceed through the use of principles and both rely on Tom Beauchamp and James Childress’s Principles of Medical Ethics for their methodology. Akabayashi and Fan refer to Beauchamp and Childress repeatedly and rarely cite positions that challenge the methodology of Principles of Medical Ethics. *(p.58)
  Response: AA is well aware that Beauchamp and Childress’s four-principle approach has received much criticism. This is why AA brought up relational autonomy or principled autonomy by O’Neill.
2. *Therefore, to attempt a principlist defense of the family-facilitated approach to informed consent, Akabayashi and Fan must either accept Beauchamp and Childress’s principle of respect for autonomy or reject this principle and redefine autonomy to encompass familial relations in East Asia. […] Despite their shared methodology, Akabayashi and Fan rank global dialogue and traditional cultural practices differently. […] Akabayashi is committed to global discourse through universal principles and Fan defends local traditions through relative principles. Their primary commitments to either the global or the local dictate whether they lean towards moral universalism or moral relativism. Their only two options are a universal principle of autonomy or a relative principle of autonomy; Akabayashi chooses the former, and Fan, the latter. *(p. 60)
  Response: AA has rejected Beauchamp and Childress’s principle of respect for autonomy and redefined autonomy to encompass familial relations. Sullivan stressed ‘in East Asia’ but AA has shown that this form of autonomy exists in the USA also, as is discussed in his 2006 *AJOB* article. Moreover, AA was critical of the opposition between moral universalism and moral relativism. AA is not a universalist. We do not know whether Ruiping Fan regards himself a relativist.
3. *Accordingly, relational autonomy cannot stand in as Akabayashi’s theory of autonomy because it cannot account for East Asian practices. Akabayashi then appeals to Onora O’Neill’s conception of “principled autonomy,” where he takes the crucial point to be the absence of coercion. However, it is hard to assess whether the appropriate safeguards for ruling out coercion are in place without further analysis of medical decision-making in Japan, as many of the respondents to his 2006 article argue. So principled autonomy cannot be Akabayashi’s theory of autonomy either, since its application is unclear in the Japanese context. In the end, Akabayashi has no universal theory of autonomy by which to justify his claims. *(p. 61)
  Response: It is correct to say that AA has no universal theory of autonomy. However, a question remains: is there a universal theory of autonomy that exists in current bioethical or philosophical discussions? For example, Kantians claim that their argument is universaliable, although they have received much criticism.
4. *Neither Akabayashi nor Fan successfully justifies East Asian informed consent. This is not because the practices they describe violate respect for autonomy, as many critics have suggested. Nor is it because they have misinterpreted the theory of moral generalism or because the theory of moral generalism is necessarily flawed. Rather, the failure of these two arguments highlights a practical problem with how bioethical discourse is pursued across cultures. Akabayashi and Fan both assume that ethical justification must be attempted according to a moral generalist methodology— principlism […]*
  *However, this assumption narrows attempted justifications: (1) it focuses the justification on the definition of ethical principles rather than a detailed description of the practices in question, and (2) it requires an explanation of the chosen principles in terms of either moral universalism or moral relativism.* (p.62)
  Response: AA does not assume that ethical justification must be attempted according to a moral generalist methodology—that is, principlism. AA asserts that the moral particularism methodology has an affinity with moral relativism, which AA wants to reject. Moreover, why does Sullivan state that ‘it requires an explanation of the chosen principles in terms of either moral universalism or moral relativism’? Why does one have to choose either universalism or relativism? If AA chooses the position of moral universalism, will the justification therefore be successful? In the next section, AA states his view regarding the conflict between moral universalism and moral relativism.
5. *For Akabayashi, the relevant principle is a form of the universal principle of autonomy, the content of which is the obligation to prevent paternalism and respect patient preferences. […] They make their arguments in terms of these principles alone and do not consider other potentially relevant moral properties. As a result, outsiders to these cultures cannot determine whether these practices should be allowed or forbidden, and insiders become distracted by a seemingly irresolvable opposition between universal and relative principles. *(p.62)
  Response: AA discusses relational autonomy and principled autonomy. AA concluded that the family-facilitated approach has an affinity with some conceptions of relational autonomy. Moreover, Sullivan stated, ‘For Akabayashi, the relevant principle is a form of the universal principle of autonomy’. However, this term was not used, and the explanation was not clear.
6. *One might counter that the failures of Akabayashi’s and Fan’s arguments should not be blamed on moral principlism because they incorrectly use principlist methodology. […] it is possible that East Asian informed consent practices indicate that a change to the principle of autonomy is necessary. In fact, revision to the principle of autonomy is exactly the argumentative route taken by both Akabayashi and Fan— Akabayashi seeks out alternative conceptions of autonomy, and Fan defines a Confucian form of autonomy. Yet while revision to principles is theoretically possible, it seldom occurs in practice. *(p.63)
  Response: AA is, once again, not using principlist theory. AA agrees with the statement, “[Y]et while revision to principles is theoretically possible, it seldom occurs in practice”. What, then, is the purpose of the revision of the principle?
7. *In the cases of Akabayashi and Fan, Akabayashi justifies his form of the principle of autonomy by tethering it to Western theories of autonomy, which he presumes to be theoretically secure. […] The requirement that the principles themselves be justified by moral theory forces Akabayashi and Fan to defend universalism and relativism, respectively. […] Within principlist ethical justification, there is no final reason why universalism or relativism is justified; the two positions are left in a deadlock.*(p.67)
  Response: AA wants to know why Sullivan states, “which he presumes to be theoretically secure”. AA did not tether autonomy because it is secure. AA does not understand why he has to defend universalism. AA agrees that the two positions are left in a deadlock. This will be discussed in the next section.
8. *What is valuable, then, is precisely what is absent from many theoretical accounts of autonomy: concrete particulars. Akabayashi’s appeal to the principle of autonomy is at best a distraction and at worst damaging to the practice. *(p.69)
  Response: AA wants to know why his appeal to the principle of autonomy is, at best, a distraction and, at worst, damaging to the practice. Is she supportive of particularism?
9. *A set of reasons such as “Japanese physicians know their patients personally, know whether patients expect them to include the family in decision-making, have communication training with families, and Japanese patients have a higher likelihood of adverse psychosocial effects following disclosure of a terminal diagnosis” would better approximate a particularist justification of some Japanese physicians’ informed consent practices, although more detailed consideration would be necessary. However, this does provide a general sketch of what a moral particularist justification might look like and what types of questions should be asked in ethical justification across cultures. Understanding ethical justification in terms of the moral particularism described in this section avoids opposition between universalism and relativism and clarifies the complex of reasons underlying particular decisions and practices. Had Akabayashi and Fan more thoroughly examined the different reasons for East Asian practices of informed consent, including those outside the scope of the principle of autonomy, they would have increased cross-cultural understanding of these practices while avoiding the competing positions of universalism and relativism. *(p.73)
  Response: AA asks whether Sullivan believes that moral particularism solves the conflict between universalism and relativism. He asks: how would moral generalists respond?

In a different article, Sullivan (2016b, 441) stated the following, citing papers by Akabayashi and Slingsby (2006), Akabayashi and Hayashi (2014a), and Fetters (1998):

*In the Japanese context, however, many find these non-disclosures to be defensible. To counter arguments against these so-called* *paternalistic actions, some attempt to define a particular Japanese type of autonomy*,* such that the physicians’ non-disclosures can be described as respecting* *patients’ autonomy via* *a* concept of family autonomy.

AA’s two papers **never** used the term ‘family autonomy.’ In this regard, Sullivan is mistaken. Cross-cultural bioethical discussions are complicated, and this is not emphasised by Sullivan. We discuss this in the next section.

## Towards Understanding Others: Bioethics Across the Globe

Most scholars say (international) dialogue is important in bioethics. There are international bodies such as the UNESCO International Bioethics Committee. Nonprofit organisations such as the Global Bioethics Initiative are active. A journal, a handbook, and an encyclopaedia of global bioethics have been published. However, global bioethics has not evolved simply or in a linear way.

It is important to understand the complexity of global bioethics. Although I have taken the East and the West as examples, even within East Asia, there are national variations, for example, between China, Japan, and Korea. In addition, different cultures exist within each nation or region. When AA refers to cross-cultural dialogue, he is referring to all of these complex relationships.

In a book published in 2020, AA wrote about the rather dark side of Japan to promote the readers’ understanding. Quoting his introduction to *The Future of Bioethics* (Akabayashi 2014),  Akabayashi (2020, ix) stated:

*Most studies in bioethics advocating East–West dialogue have either attempted cross-cultural comparison or proposed Eastern philosophical paradigms as a counter to Western ideas.* *The tacit premise of previous writing on East–West dialogue is therefore a strain of relativism. From the Eastern perspective, Western views are treated as a cultural construct that should be referenced as models, but are not appropriate to be utilized in their existing form.* *To Westerners, Eastern interpretation represents ways of thinking that should be recognized but can never truly be understood in their complexity within Western cultures*. *For this reason, Asians place Western conceptions of bioethics on the critical chopping block, and approach them as something to be overcome. In contrast, although Westerners occasionally comment on current conditions in Asian countries, they rarely fully engage with bioethical discussions led by Asian researchers, and neither express agreement nor fully critique such views. In a globalized world, simply maintaining a respectful distance from other cultures is no longer sufficient.*

Akabayashi (2020, x) stated the aim of the single-author monograph in the introduction as follows:

*As many scholars have proposed, a dialogue that encourages both local and global thinking is needed. I believe that such a dialogue must be enabled by mutual understanding or, at the very least, a healthy attitude and sincere effort toward obtaining it. This book is intended to serve as a tool to promote this […] this book is* *open access*. […]. *Thus, readers from low to middle income countries as well as students and laypersons can read them for free. I urge all who read this book to write their own story for their own country to add to this dialogue. In this regard, this is not an introductory book on Japan, but rather the beginning of the series comprising many other narratives to come which will serve as tools that will facilitate the international and mutual* *understanding* *we require to initiate genuine dialogue.*

When discussing the deadlock between universalism and relativism AA stated that “universalism versus relativism is a long-standing debate in Western philosophy. However, in the 50 years in which global bioethics has evolved, the discussion has not moved forward in a meaningful way. In general, it appears that Western philosophers have agreed that the radical extremes (extreme universalism and extreme relativism) should be discarded, but that is all” (Akabayashi 2020, 120)*.* Akabayashi (2020, 121) continued “[L]et us set aside the opposition between universalism and relativism in order to create a new framework, for if arguments pertaining to global bioethics continue in the same direction, then we will be forever stuck in a dead-end conversation”. Finally, Akabayashi (2020, 122) stated the following:

*I also have a radical proposal. Namely, that we discard the term “global bioethics.” This is not a proposal to set aside this term temporarily, with the potential for reuse later on, but rather to permanently reject it. As well, when using terms like “Asian Bioethics” and “African* *Bioethics,”* *the adjectives “Asian” and “African” also signal relativism*. *[…]* *Therefore, I propose we use *“***Bioethics Across the Globe (BAG)***”* in place of “global bioethics.” While I feel that this term (global bioethics) embodies a more universal tone, I also wonder if some would interpret the adjective “global” to mean that BAG is no longer trying to achieve universalism*. […]*. (However,) at the very least, BAG is preferable to the current term, “global bioethics.” The time has come to discard the term “global bioethics,” which will forever imply the opposition between universalism and relativism. *(Author's emphasis).

We were highly motivated to respond when we found Sullivan’s article (2016a). We believe that her criticism of AA is based upon misunderstanding. Although Sullivan’s paper focuses on bioethical discourse across cultures, proposing ways to enable cross-cultural dialogue to be pursued more productively, her discussion is often based upon misinterpretation. This is essential to move dialogue further.

AA has been criticised because he did not fully explain or define the concept of BAG. He attests that in fact a firm definition is not possible at this time. This is consistent with the view of the founder of modern bioethics, Van Rensselaer Potter (1971, 1988, 1990) when he discussed the initial development of the term ‘bioethics.’ Both bioethics and now global bioethics signify new ways of thinking. Both are dependent on an in-depth understanding of others. This is the spirit of BAG.

## Concluding Remarks

Beginning with an analysis of the nature of autonomy in Japan, we went on to discuss the opposition between universalism and relativism. It is our view that the two positions remain deadlocked.

Once again, we hope that those who read this article will try to engage in works to facilitate an in-depth understanding of all countries and societies in their diversity. When we can come together to engage with these issues a real dialogue is possible. We want to use this engagement to go on to develop other projects, in particular, an anthology on the ethics of war. We contend that understanding the nature of autonomy in different cultures will be invaluable for a discussion about the ethics of war and through this, human survival. Autonomy is closely related to human beings’ intrinsic value, self, authenticity, dignity, and capacity, all fundamental to human flourishing. As bioethics researchers, we should fully understand our mission and then pass over this spirit to the next generation.
